# Patient-reported outcome measures for masticatory function in adults: a systematic review

**DOI:** 10.1186/s12903-021-01949-7

**Published:** 2021-11-23

**Authors:** Yanpin Fan, Xin Shu, Katherine Chiu Man Leung, Edward Chin Man Lo

**Affiliations:** Faculty of Dentistry, The University of Hong Kong, 1/F, Prince Philip Dental Hospital, 34 Hospital Road, Sai Ying Pun, Hong Kong, China

**Keywords:** Mastication, Chewing ability, Patient reported outcome measures, Epidemiology, Adult

## Abstract

**Objective:**

The aim of this systematic review was to critically evaluate the Patient-Reported Outcome Measures (PROMs) for masticatory function in adults.

**Methods:**

Five electronic databases (Medline, Embase, Web of Science Core Collection, CINAHL Plus and APA PsycINFO) were searched up to March 2021. Studies reporting development or validation of PROMs for masticatory function on adults were identified. Methodological quality of the included studies was evaluated using the COnsensus-based Standards for the selection of health Measurement INstruments (COSMIN) risk of bias checklist. Psychometric properties of the PROM in each included study were rated against the criteria for good measurement properties based on the COSMIN guideline.

**Results:**

Twenty-three studies investigating 19 PROMs were included. Methodological qualities of these studies were diverse. Four types of PROMs were identified: questions using food items to assess masticatory function (13 PROMs), questions on chewing problems (3 PROMs), questions using both food items and chewing problems (2 PROMs) and a global question (1 PROM). Only a few of these PROMs, namely chewing function questionnaire-Chinese, Croatian or Albanian, food intake questionnaire-Japanese, new food intake questionnaire-Japanese, screening for masticatory disorders in older adults and perceived difficulty of chewing-Tanzania demonstrated high or moderate level of evidence in several psychometric properties.

**Conclusions:**

Currently, there is no PROM for masticatory function in adults with high-level evidence for all psychometric properties. There are variations in the psychometric properties among the different reported PROMs.

*Trial Registration* PROSPERO (CRD42020171591).

**Supplementary Information:**

The online version contains supplementary material available at 10.1186/s12903-021-01949-7.

## Background

Masticatory difficulty or masticatory problem is prevalent in older adults worldwide [1–3]. Masticatory function has been found to be associated with physical activity level, disability, comorbidities and cognitive status [4]. A recent consensus report classified the methodologies for assessment of masticatory function into three types, namely direct objective assessment, indirect assessment and subjective assessment [5]. In direct objective assessment, masticatory function is evaluated by assessing a test material either after a pre-determined number of chewing strokes (masticatory performance) or at the moment when the study participant feels the urge to swallow (swallowing threshold). In indirect objective assessment, masticatory function is evaluated by jaw kinematics, muscle activity, tongue or lip function, and saliva secretion. In subjective assessment, self-assessment of masticatory function is evaluated using questionnaires and interviews. A recent systematic review reported that none of the established objective assessments of masticatory function had strong evidence for all measurement properties and these assessments required sieves or digital image software [6].

Self-assessment of masticatory function uses patient-reported outcome measures (PROMs), mainly questionnaires. This has the advantage of assessing masticatory function from the person’s perspective, taking into account adaptational and psychological factors. Some studies found there were correlations between self-assessment and objective assessment of masticatory function [7–9]. However, other researchers reported a lack of agreement between the subjective and the objective assessments of masticatory function [10, 11]. It should be noted that not all PROMs are created equal, and well-designed PROMs are needed to reveal the true masticatory function. Quality of the information collected and strength of the conclusion made depend on the properties of the instrument used in the study [12]. The methodological quality and psychometric properties of PROMs, such as content validity, structural validity, reliability, internal consistency and construct validity, are important aspects for the development or selection of a reliable and valid measurement tool [13, 14]. It is important to use PROMs which have undergone rigorous psychometric testing to ensure the results obtained are valid and reliable. The COSMIN guideline was developed to enhance the quality of systematic review of PROMs [15–18].

In the past decade, a systematic review of all generic PROMs for adult dental patients [19] and a few systematic reviews of PROMs used in implant dentistry [20–22] were published. However, to the best of our knowledge, there is no systematic review of PROMs for assessing masticatory function. Therefore, based on the COSMIN guideline, this review aimed to identify PROMs that have been used in adults (population) for subjective assessment (type of instruments) of masticatory function (construct), and to evaluate the methodological qualities and psychometric properties (measurement properties of interest) of these PROMs.

## Methods

This systematic review was registered in PROSPERO (Registration Number: CRD42020171591), and was reported according to the Preferred Reporting Items for Systematic Reviews and Meta-Analyses (PRISMA) 2020 Checklist [23].

### Search strategy

Medline (Pubmed), Embase (Ovid), Web of Science Core Collection, CINAHL Plus (EBSCOhost) and APA PsycINFO (ProQuest) were searched from their inception to March 2021. The search strategy consisted of three parts: (1) “chewing function/ability” or “masticatory function/ability” or mastication; (2) questionnaire* or subjective* or evaluation* or assessment*; and (3) validation or validity or reliability or psychometric*. The detail search strategies can be found in Additional file 1: Part 1. As a supplement, manual search on the reference lists of published reviews and the included articles, and the Google Scholar was performed.

### Eligibility criteria

Based on the COSMIN guideline, the study inclusion criteria in the present review were: (1) studies investigating the development or validation (measurement properties of interest) of subjective assessment (type of instruments) of masticatory function (construct), regardless of study design; (2) studies on adults (population); and (3) studies published in English with full text available.

The study exclusion criteria were: (1) studies which only included objective assessment of masticatory function; (2) studies that used subjective assessment of masticatory function as an outcome measure only; and (3) case studies, expert opinion, animal studies and reviews.

### Study selection and data extraction

Articles retrieved from the electronic search were imported into the EndNote reference program (Ver. 9.3.1). After removing duplicates, two reviewers (YPF and XS) independently screened the titles and the abstracts of all identified records, and evaluated the full texts of all potentially eligible articles. The following data were extracted from the included articles: first author, year of publication, study participants, study setting, study design, study location, and the characteristics and psychometric properties of PROMs. Any disagreements between the two reviewers were resolved by discussion with an expert researcher (ECML).

### Evaluation of the methodological quality of each study

Methodological quality of the included studies was evaluated using the COSMIN risk of bias checklist [15]. Following the COSMIN manual for systematic reviews of PROMs and the COSMIN methodology for evaluating content validity [16, 18], all procedures were conducted by two reviewers (YPF and XS) independently. The COSMIN risk of bias checklist included 10 aspects: PROM development, content validity, structural validity, internal consistency, cross-cultural validity/measurement invariance, reliability, measurement error, criterion validity, hypotheses testing for construct validity, and responsiveness. The methodological quality of each aspect was assessed and rated on a 4-point scale: “very good” (V), “adequate” (A), “doubtful” (D), and “inadequate” (I). The ratings were determined based on “the worst score counts” principle, i.e. the lowest rating for any item was the rating for the study [24].

### Evaluation of the quality of psychometric properties

Psychometric properties of the PROM in each included study were rated against the criteria for good measurement properties [14, 25]. Each property was rated as sufficient (+), insufficient (−) or indeterminate (?). After assessing the quality of the psychometric properties of the PROM in the different studies, the quality of each psychometric property of the PROM was rated as sufficient (+), insufficient (−), inconsistent (±) or indeterminate (?). Finally, the level of evidence of each psychometric property of that particular PROM was graded as “high”, “moderate”, “low” or “very low”, using a modified Grading of Recommendations Assessment, Development, and Evaluation (GRADE) approach recommended in the COMSIN guideline [16]. According to the COSMIN guideline, publication bias was not considered when using the modified GRADE to evaluate the measurement properties of PROMs, and only the following four factors were evaluated: risk of bias, inconsistency, imprecision and indirectness.

## Results

### Study selection

**Fig. 1:**
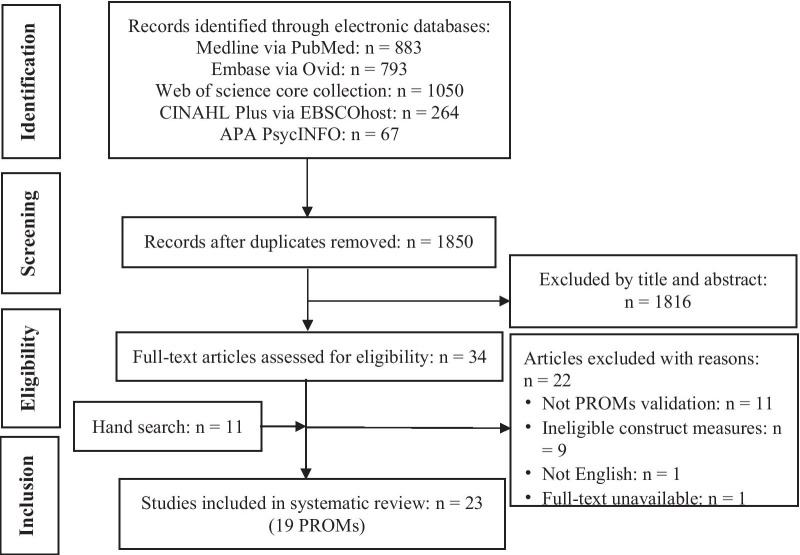
Flow Diagram, March 2021

After removal of duplicates, 1850 records were identified (Fig. 1). The titles and abstracts of these records were screened, and 1816 records were excluded. Full texts of 34 articles were assessed and 22 articles were excluded with reasons. Eleven articles were included through the supplementary search (Additional file 1: Part 2). Finally, 23 articles met the inclusion and exclusion criteria.

### Characteristics of the included studies and PROMs

Summary of the 23 included articles reporting on 23 studies and 19 PROMs is presented in Table 1. The total number of participants across all studies was 16,886 and the participants were from all adult age groups. There were 20 cross-sectional studies and three cohort studies. Among these studies, eight were conducted in Japan, four in China, two in Canada, one each in Spain, Iran, Brazil, Croatia, the Republic of Kosovo, Korea, Sudan, Tanzania, and Sweden. The sample size ranged from 20 to 2244. There were four types of PROMs: (1) questions related to chewing specific food items (15 studies, 13 PROMs) [7, 8, 26–38], (2) questions related to chewing problems (three studies, three PROMs) [39–41], (3) questions related to chewing specific food items and questions related to chewing problems (three studies, two PROMs) [42–44], and (4) a global question (two studies, one PROM) [45, 46].

**Table 1:** Characteristics of the included studies

PROMs-abbreviation	PROMs-full name	Author, year of publication	Study participant	Study setting	Study design	Study location	PROM language	Number of items	Item characteristics	Response options	Summary sore (Yes-range/No)	Intended construct and domains
Food items												
CFS	Chewing function score [31] (Development)	Sato et al., 1989	110 older adults; mean age: 71.6 years; female: 58.2%; sample: random	Selected complete denture wearers in dental clinics	Cross-sectional	Japan	Japanese	20	20 food items	Easy (1), difficult (0), impossible (0) to chew	Yes, 0–20	Chewing function, unidimensional
ICA-1990	Index of chewing ability-1990 [32]	Leake, 1990	233 older adults	Independently living elders (secondary data)	Cross-sectional	Canada	English	5	9 food items, 5-item index	Yes (1) or No (0)	Yes, 0–5	Chewing ability, unidimensional
ICA-2020	Index of chewing ability-2020 [29]	Montero et al., 2020	118 adults; age: 30–86 years; female: 41.5%;	Patients who required rehabilitation in dental hospital	Cohort	Spain	Spanish		5-item index	0 = no difficulty, 1 = little, and 2 = much difficulty	-	
FIQ-Japanese-1994	Food intake questionnaire Japanese-1994 [7]	Hirai et al., 1994	20 older adults; age: 50–76 years; female: 45%	Complete denture wearers	Cross-sectional	Japan	Japanese	35	35 food items	Easily eaten (2), eaten with difficulty (1), “cannot be eaten” or “do not eat” (0)	Yes, weighted score, 0–100%	Masticatory function
FIQ-Japanese-1998	Food intake questionnaire- Japanese-1998 [33]	Miura et al., 1998	70 older adults; age: 65–74 years; female: 51.4%	Residents living in Kawaguchiko, Yamanashi Prefecture	Cross-sectional							
New-FIQ- Japanes	New Food intake questionnaire-Japanese [8]	Koshino et al., 2008	262 older adults; mean age: 76.7 years; female: 51.1%	Complete denture wearers	Cross-sectional	Japan	Japanese	25	25 food items	Easily eaten (2), eaten with difficulty (1), “cannot be eaten” or “do not eat” (0)	Yes, weighted score, 0–100%	Masticatory function, 5 factors, namely hardness, fibrous, slippery, sticky, other factor
FIQ-Chinese-2012	Food intake questionnaire-Chinese-2012 [38]	Hsu et al., 2012	2244 dental patients; age: 45 + years; female: 51.5%	Patients recruited from dental clinics	Cross-sectional	China, Taiwan	Chinese	14	14 food groups	2 = able to eat, 1 = difficult to eat, 0 = unable to eat	Yes	Masticatory ability
FIQ-Chinese-2014	Food intake questionnaire-Chinese-2014 [28]	Hsu et al., 2014	332 elders; age: 65 + years; female: 52.1%; sample: convenient	Elders recruited from senior citizens’ service center, community dwelling	Cross-sectional					‘easy to chew,’ ‘difficult to chew,’ or ‘unable to chew’		
PDC-Tanzania	Perceived difficulty of chewing- Tanzania [34]	Sarita et al., 2003	850 subjects; age: 20 + years; sample: convenient	725 subjects with shortened dental arch and 125 subjects with complete dental arch living in urban and rural areas	Cross-sectional	Tanzania	Tanzanian	20	20 food items	0 = very easily; 1 = minor problems, adapted; 2 = minor problems, not adapted; 3 = difficult but not avoided; 4 = very difficult but not avoided; 5 = very difficult and avoided; 6 = never used that food	Yes, mean score, 0–5	Difficulty of chewing
PDC-Sudan	Perceived difficulty of chewing-Sudan [26]	Khalifa et al., 2013	1,888 individuals; age:16 + years; female: 59%; sample: consecutive, proportional sampling	Patients attending outpatient dental hospitals (n = 1659) and dental health centres (n = 229)	Cross-sectional	Sudan	Sudanese	15	15 food items	0 = very easy; 1 = minor problems, adapted; 2 = minor problems, not adapted; 3 = difficult but not avoided; 4 = very difficult but not avoided; 5 = very difficult and avoided; 6 = have never eaten that food	Yes, 0–15	Difficulty of chewing
IED	Index of eating difficulty [35]	Zeng et al., 2008	1229 participants; age: 55 + years; female: 51.7%; sample: convenient	Elders attending check-up centre for annual health screening	Cross-sectional	China	Chinese	10	5 food groups (10 food items)	Yes or No	Yes, 0–5	Eating difficulty, unidimensional
CFQ-Japanese	Alternate version of the chewing function questionnaire [36]	Baba et al., 2009	491 subjects, mean age: 63.0 years; female: 71%; sample: consecutive	Partially dentate patients who attended the prosthodontic clinic	Cross-sectional	Japan	Japanese	20	20 food items	Easy (1) or difficult (0) to chew the foods	Yes, 0–20	Chewing function, unidimensional, one construct
CFQ-Chinese	Chewing function questionnaire-Chinese [30]	Fan et al., 2021	211 elders; mean age: 77.1 years, female: 69.7%; sample: purposive	Elders from a dental hospital, an elderly home and three community elderly centres	Cross-sectional	China	Chinese	10	10 food items	Impossible (1), difficult (2), easy (3) to chew	Yes, 10–30	Chewing function, unidimensional
FIAQ	Food intake ability questionnaire [37]	Kim et al., 2009	308 adults; age: 20 + years; female: 63.6%	Patients in dental hospitals	Cross-sectional	Korea	Korean	30	30 food items	Cannot chew at all (1), difficult to chew (2), cannot either way (3), can chew some (4), can chew well (5)	Yes, average score, 1–5	Masticatory function
FIAQ-key food version	Food intake ability questionnaire- key food version [37]	Kim et al., 2009	308 adults; age: 20 + years female: 63.6%	Patients in dental hospitals	Cross-sectional	Korea	Korean	5	5 food items	Cannot chew at all (1), difficult to chew (2), cannot either way (3), can chew some (4), can chew well (5)	Yes, average score, 1–5	Masticatory function
MACE	Masticatory ability assessment for community-dwelling elderly [27]	Miura et al., 2013	761 elderly subjects; age: 65–84 years; female: 55.1%;	Independent community-dwelling elders	Cross-sectional	Japan	Japanese	9	9 food items	Easy (2), slightly difficult (1), very difficult (0), nonresponse	Yes, 0–18	Masticatory ability, unidimensional
Chewing problems												
MPI	Masticatory problem index [39]	Tsuga et al., 1998	160 elders; age: 80-year-old; female: 53.8%; sample: random	Elders living in their own homes, not institutionalized and could travel to the clinical examination	Cross-sectional	Sweden	Swedish	13	13 items related to chewing problems	–	Yes	Masticatory problem
Subset-OHIP	Subset of the oral health impact profile [40]	Cusson et al., 2015	1,789 older adults; age: 67–84 years; sample: random	Community-dwelling adults	Cross-sectional	Canada	English	7	7 items related to chewing problems	Always, often, occasionally, rarely, never	Yes, 0–28	Masticatory efficiency, unidimensional
SMDOA	Screening for masticatory disorders in older adults [41]	Cavalcanti et al., 2019	295 older adults; age: 60 + years; female: 86.4%; sample: convenient	Adults in social centers for epidemiological screening purpose	Cross-sectional	Brazil	Portuguese	9	9 items related to chewing problems	No, Yes-sometimes, Yes-always	No. Detect change in chewing function	Masticatory disorders, unidimensional. Two factors: 1. chewing function 2. masticatory perception
Food items and chewing problems												
CFQ-Croatian	Chewing function questionnaire-Croatian [44]	Peršić et al., 2013	224 participants; age:19–85 years; female: 48.0% sample: convenient	Dental students and prosthodontic patients for daily clinical practice and research	Cohort	Croatia	Croatian and English	10	10 items, including food items and items related to chewing problems	0 = never, 1 = hardly ever, 2 = occasionally, 3 = fairly, 4 = very often or extreme difficulties	Yes, 0–40	Chewing function, unidimensional
CFQ-Albanian	Albanian version of chewing-function questionnaire [43]	Bimbashi et al., 2016	205 subjects; age:19–86 years; female: 53.2% sample: random (36), consecutive (61), convenient (108)	General population, dental students and prosthodontic patients for research and clinical trials	Cohort	Republic of Kosovo	Albanian					
QMFQ-Persian	Persian version of the quality of masticatory function questionnaire [42]	Khodaeian et al., 2016	62 edentulous patients; age: 45–75 years; female: 50%; sample: convenient	Complete denture wearers referred from hospitals	Cross-sectional	Iran	Persian	27	27 items, including food items and items related to chewing problems	“always = 1” to “never = 5” or “a lot = 1” to “no difficulty = 5”	Yes, 27–135	Masticatory function, five domains: masticatory problems with dentures, problems while consuming apple and carrot, meat products, fruits and vegetables, and changes need for better swallowing
One global question												
SMF-Yanagisawa	Self-reported masticatory function [45]	Yanagisawa et al. 2010	2668 adults; age: 40–75 years; female: 59.1%; sample: convenient	Community residents recruited by mail	Cross-sectional	Japan	Japanese	1	1 global question. “Can you bite tightly with your back teeth and dentures?”	Yes, I can bite tightly on both sides; Yes, but only on one side; No, I cannot on either side	No	Masticatory function
SMF-Ueno	Self-reported masticatory status (function) [46]	Ueno et al., 2018	2356 adults; age: 40–75 years; female: 67.9%; sample: convenient	Community residents recruited on-site or by mail	Cross-sectional	Japan	Japanese					

The number of questions in each PROM ranged from seven to 35. Most of the PROMs were unidimensional, except the Persian version of the quality of masticatory function questionnaire (QMFQ-Persian) which contained five domains [42]. Variations were found in the response options of the included PROMs. For the three PROMs with questions about chewing problems, one adopted a five-point Likert-scale (“always”, “often”, “occasionally”, “rarely” and “never”) [40], one provided three choices (“No”, “Yes-sometimes” and “Yes-always”) [41], while the other accepted different responses for different questions [39]. For the PROMs containing questions about chewing specific food items and questions about chewing problems, all adopted a five-point Likert scale response option [42–44]. For the PROM containing only one global question, the response choices were “Yes, I can bite tightly on both sides”, “Yes, but only on one side” and “No, I cannot bite on either side” [45, 46]. For the PROMs with questions about chewing specific food items, the response options were based on level of difficulties with slight variations.

### Methodological quality of each study

An overview of the methodological quality assessment of the included studies is presented in Table 2. Nearly all (21 out of 23) studies had conducted hypothesis testing for structural validity and their methodological qualities were rated as adequate or very good. Most of the studies that evaluated internal consistency were rated as doubtful because information on structural validity or unidimensionality of PROMs was not presented [26–28, 32, 34, 35, 38, 40]. For the studies that evaluated structural validity, most studies were rated as adequate or very good, except for the study reporting on QMFQ-Persian, which was rated as inadequate due to insufficient sample size [42]. Of the three studies that evaluated criterion validity, two studies were rated as very good [8, 27], and one was rated as inadequate [37]. Only three studies evaluated responsiveness and their methodological qualities were rated as very good [29, 43, 44]. Regarding cross-cultural validity, two studies were rated as doubtful [42, 43]. Among the six studies which had evaluated content validity, five were rated as doubtful [35, 41–44] while one study was rated as inadequate [30]. Regarding PROM development, six studies described the development process and the methodological qualities of all these studies were rated as doubtful [30, 31, 35, 38, 41, 44].

**Table 2:** Methodological quality of the included studies

PROMs	Internal consistency	Test–retest reliability	Content validity	Structural validity	Criterion validity	Hypothesis testing for construct validity		Responsiveness	Cross-cultural translation/validity
						Convergent validity	Discriminative validity		
Food items									
CFS [31]	0	0	0	0	0	0	0	0	0
ICA-1990 [32]	D	0	0	0	0	A	0	0	0
ICA-2020 [29]	0	0	0	0	0	0	0	V	0
FIQ-Japanese-1994 [7]	0	0	0	0	0	V	0	0	0
FIQ-Japanese-1998 [33]	0	0	0	0	0	A	0	0	0
New-FIQ-Japanese [8]	D	0	0	A	V	V	0	0	0
FIQ-Chinese-2012 [38]	D	D	0	0	0	0	V	0	0
FIQ-Chinese-2014 [28]	D	D	0	0	0	0	A	0	0
PDC-Tanzania [34]	D	0	0	0	0	0	A	0	0
PDC-Sudan [26]	D	A	0	0	0	A	0	0	0
IED [35]	D	D	D	0	0	A	0	0	0
CFQ-Japanese [36]	V	A	0	A	0	A	0	0	0
CFQ-Chinese [30]	V	A	I	A	0	A	A	0	0
FIAQ [37]	0	0	0	A	0	A	A	0	0
FIAQ-key food [37]	0	0	0	0	I	A	A	0	0
MACE [27]	D	0	0	0	V	A	0	0	0
Chewing problems									
MPI [39]	0	0	0	0	0	A	0	0	0
Subset-OHIP [40]	D	0	0	0	0	V	0	0	0
SMDOA [41]	0	0	D	V	0	0	V	0	0
Food items and chewing problems									
CFQ-Croatian [44]	V	V	D	A	0	A	V	V	0
CFQ-Albanian [43]	V	V	D	A	0	A	V	V	D
QMFQ-Persian [42]	V	0	D	I	0	A	0	0	D
One global question									
SMF-Yanagisawa [45]	0	0	0	0	0	A	0	0	0
SMF-Ueno [46]	0	0	0	0	0	A	0	0	0

V, very good; A, adequate; D, doubtful; I, inadequate; 0, no data available

### Quality of psychometric properties

Psychometric properties of the PROMs in the individual studies are presented in Table 3. The details can be found in Additional file 1: Part 3. Internal consistency was evaluated in 14 studies, and nine of them were rated as indeterminate, because these studies did not meet the criteria “at least low evidence for sufficient structural validity”. Eight studies evaluated test–retest reliability and seven of them were rated as sufficient. Content validity was evaluated in six studies and four of them were rated as sufficient. Eight studies evaluated structural validity and seven of them were rated as sufficient. Only three studies reported criterion validity and two of them were rated as sufficient. Twelve of the 21 studies that had conducted hypothesis testing for construct validity were rated as sufficient. All of the three studies that evaluated responsiveness were rated as sufficient, because the standardized effect size was higher than expected and the results were in accordance with the hypothesis. Two studies performed cross-cultural translation, and both were rated as indeterminate.

**Table 3:** Psychometric properties of the included PROMs

PROMs	Internal consistency	Test–retest reliability	Content validity	Structural validity	Criterion validity	Hypothesis testing for construct validity	Responsiveness	Cross-cultural translation/validity
Food items								
CFS [31]	0	0	0	0	0	0	0	0
ICA-1990 [32]	?	0	0	0	0	?	0	0
ICA-2020 [29]	0	0	0	0	0	0	+	0
FIQ-Japanese-1994 [7]	0	0	0	0	0	+	0	0
FIQ-Japanese-1998 [33]	0	0	0	0	0	+	0	0
New-FIQ-Japanese [8]	?	0	0	+	+	+	0	0
FIQ-Chinese-2012 [38]	?	+	0	0	0	+	0	0
FIQ-Chinese-2014 [28]	?	+	0	0	0	–	0	0
PDC-Tanzania [34]	?	0	0	0	0	+	0	0
PDC-Sudan [26]	?	+	0	0	0	?	0	0
IED [35]	?	+	?	0	0	–	0	0
CFQ-Japanese [36]	+	–	0	+	0	–	0	0
CFQ-Chinese [30]	+	+	?	+	0	+	0	0
FIAQ [37]	0	0	0	?	0	–	0	0
FIAQ-key food [37]	0	0	0	0	?	+	0	0
MACE [27]	?	0	0	0	+	–	0	0
Chewing problems								
MPI [39]	0	0	0	0	0	–	0	0
Subset-OHIP [40]	?	0	0	0	0	–	0	0
SMDOA [41]	0	0	+	+	0	+	0	0
Food items and chewing problems								
CFQ-Croatian [44]	+	+	+ ?	+	0	+	+	0
CFQ-Albanian [43]	+	+	+ ?	+	0	+	+	?
QMFQ-Persian [42]	+	0	+	+	0	–	0	?
One global question								
SMF-Yanagisawa [45]	0	0	0	0	0	+	0	0
SMF-Ueno [46]	0	0	0	0	0	+	0	0

The hypothesis for evaluating convergent validity was if a correlation between the PROM under study and the comparator instrument measuring the similar construct was ≥ 0.50, it was considered as sufficient [60]. The hypothesis testing for evaluating discriminant validity and responsiveness were in accordance with that in individual studies

+  = sufficient; –= insufficient; ? = indeterminate; 0 = no data available

### Evidence synthesis

Summarized evidence of the included PROMs is presented in Table 4. The levels of evidence differed amongst the various psychometric properties of the PROMs. Chewing Function Questionnaires-Croatian or Albanian (CFQ-Croatian or Albanian) had a moderate or high level of evidence for internal consistency, test–retest reliability, structural validity, hypothesis testing for construct validity and responsiveness, and these psychometric properties were all rated as sufficient [43, 44]. Food Intake Questionnaire-Japanese (FIQ-Japanese) had moderate level of evidence for sufficient hypothesis testing for construct validity [7, 33]. New Food Intake Questionnaire-Japanese (New-FIQ-Japanese) had a moderate or high level of evidence for structural validity, criterion validity and hypothesis testing for construct validity, and these psychometric properties were all rated as sufficient [8]. Perceived Difficulty of Chewing-Tanzania (PDC-Tanzania) had moderate level of evidence for sufficient hypothesis testing for construct validity and low level of evidence for indeterminate internal consistency [34]. Chewing Function Questionnaire-Chinese (CFQ-Chinese) had high level of evidence for internal consistency, and moderate level of evidence for test–retest reliability, structural validity and hypothesis testing for construct validity, and these psychometric properties were all rated as sufficient [30]. Screening for Masticatory Disorders in Older Adults (SMDOA) had high level of evidence for structural validity and hypothesis testing for construct validity, and low level of evidence for content validity, and these psychometric properties were all rated as sufficient [41].

**Table 4:** Evidence synthesis of the included PROMs

PROMs	Internal consistency	Test–retest reliability	Content validity	Structural validity	Criterion validity	Hypothesis testing for construct validity	Responsiveness	Cross-cultural translation/validity
Food items								
CFS [31]	0	0	0	0	0	0	0	0
Level of evidence								
ICA [29, 32]	?	0	0	0	0	?	+	0
Level of evidence	Low					Moderate	High	
FIQ-Japanese [7, 33]	0	0	0	0	0	+	0	0
Level of evidence						Moderate		
New-FIQ-Japanese [8]	?	0	0	+	+	+	0	0
Level of evidence	Low			Moderate	High	High		
FIQ-Chinese [28, 38]	?	+	0	0	0	±	0	0
Level of evidence	Moderate	Moderate				Moderate		
PDC-Tanzania [34]	?	0	0	0	0	+	0	0
Level of evidence	Low					Moderate		
PDC-Sudan [26]	?	+	0	0	0	?	0	0
Level of evidence	Low	Moderate				Moderate		
IED [35]	?	+	?	0	0	−	0	0
Level of evidence	Low	Low	Low			Moderate		
CFQ-Japanese [36]	+	−	0	+	0	−	0	0
Level of evidence	Moderate	Moderate		Moderate		Moderate		
CFQ-Chinese [30]	+	+	?	+	0	+	0	0
Level of evidence	Moderate	Moderate	Very low	Moderate		Moderate		
FIAQ [37]	0	0	0	?	0	−	0	0
Level of evidence				Moderate		Moderate		
FIAQ-key food [37]	0	0	0	0	?	+	0	0
Level of evidence					Very low	Moderate		
MACE [27]	?	0	0	0	+	−	0	0
Level of evidence	Low				High	Moderate		
Chewing problems								
MPI [39]	0	0	0	0	0	–	0	0
Level of evidence						Moderate		
Subset-OHIP [40]	+	0	0	0	0	–	0	0
Level of evidence	High					High		
SMDOA [41]	0	0	+	+	0	+	0	0
Level of evidence			Low	High		High		
Food items and chewing problems								
CFQ (Croatian, Albanian) [43, 44]	+	+	+ ?	+	0	+	+	?
Level of evidence	High	High	Moderate	Moderate		Moderate	High	Low
QMFQ-Persian [42]	+	0	+	+	0	-	0	?
Level of evidence	Very low		Low	Very low		High		Very low
One global question								
SMF [45, 46]	0	0	0	0	0	+	0	0
Level of evidence						High		

+  = sufficient;−= insufficient; ±  = inconsistent; ? = indeterminate; 0 = no data available

## Discussion

This review yielded two major findings: (1) although 19 PROMs for masticatory function were identified, none of them had high-level evidence for all of the sufficient psychometric properties; and (2) CFQ (Croatian or Albanian), FIQ-Japanese, new-FIQ-Japanese, CFQ-Chinese, SMDOA and PDC-Tanzania have better psychometric properties than the other PROMs.

### Comparison with previous reviews

There is a recent consensus report on the assessment of masticatory function [5] in the literature. In the consensus report [5], five PROMs for masticatory function were mentioned, among which four were included in the present review. The PROM that was not included in the present review was an instrument containing three questions based on the international classification of functioning, disability and health (ICF) model for oral function [47]. The reason for not including this PROM is the development or validation of the PROM was not reported in the literature.

In a recent systematic review of PROMs for adult dental patients [19], only two out the 20 questionnaires were on masticatory function and they were included in the present review. There were three other questionnaires included in that systematic review but they were not included in the present review because they focused on jaw function and not masticatory function.

Since the methodological quality and psychometric properties of PROMs for masticatory function have not been reported in previous systematic reviews, no comparison regarding the findings from the present review and those of earlier reviews can be made.

### Recommendations on methodology and psychometric property for future research

In the present review, only six studies described the PROM development process and this was only briefly presented [30, 31, 35, 38, 41, 44]. It is hard to tell whether the PROM development process had not been properly carried out or was just not reported. Detailed information about the PROMs development process should be described in future research.

None of the 23 studies included in the present review tested the measurement errors. Measurement error is defined as “the systematic and random error of a study participant’s score that is not attributed to true changes in the construct to be measured” [48]. The measurement error will be rated as sufficient if the minimal important change (MIC) is larger than the smallest detectable change (SDC), or MIC is outside the limits of agreement (LOA). A PROM can be used to compare masticatory function of different people or the same person at different time points. The difference between two scores may originate from the measurement error or the real difference/change. Lack of assessment of the measurement error may affect judgment. Thus, the measurement error of PROMs should be evaluated in future studies in order to obtain accurate results and to draw valid conclusions.

The present review found that only two studies evaluated the cross-cultural validity of the PROMs but they only conducted forward–backward translation [42, 43]. It is not sufficient for the evaluation of cross-cultural validity by merely performing forward–backward translation or by conducting a pilot study on a sample with a different culture without carrying out proper statistical analysis. To assess cross-cultural validity of PROMs in future studies, regression analyses or confirmatory factor analysis (CFA) using classical test theory (CTT) methods, and differential item functioning (DIF) analyses using item response theory (IRT) methods are recommended [49–52].

Responsiveness is defined as “the ability to detect clinically important change” or as “the ability to detect a change in the construct to be measured” [48]. In the present review, there were only three longitudinal studies, two on CFQ (Croatian or Albanian) and one study on the Index of Chewing Ability (ICA, 2020), which evaluated the responsiveness of the PROMs through the change scores collected before and after prosthodontic treatment [29, 43, 44]. The responsiveness of the other 17 PROMs was not studied and they may not be able to detect changes in masticatory function. Therefore, further studies should be conducted to evaluate the responsiveness of PROMs. In addition, the time span needed to capture the score difference in different populations, e.g. young adults and older adults, should be taken into consideration when designing such studies. The effect sizes (mean change score/SD baseline) [53], standardized response mean (mean change score/SD change score) [54], Norman’s responsiveness coefficient (σ^2^ change/σ^2^ change + σ^2^ error) [55], and relative efficacy statistics ((t‐statistic_1_/t‐statistic_2_)^2^) [56] are appropriate statistical methods to evaluate responsiveness. In contrast, use of paired t-test is not appropriate for this purpose [57].

Content validity was only evaluated in five of the 19 PROMs included in the present review, and none of these PROMs have high level of evidence on content validity. It is worth emphasizing that content validity, defined as the degree to which the content of a PROM is an adequate reflection of the construct to be measured [48], is widely regarded as the most important type of validity for PROMs [58]. Asking study participants about the relevance, comprehensiveness and comprehensibility of a PROM, and obtaining the views of professionals about the relevance and comprehensiveness of a PROM, are essential when designing a PROM with sufficient content validity and strong level of evidence [58]. It is strongly recommended that future research can refer to the COSMIN guideline to develop all PROMs that have sufficient validity with strong level of evidence.

### Recommendations on the selection of appropriate PROMs for future research

The PROMs included in this review were put into three categories based on the COSMIN manual [16]. Category A includes PROMs with evidence for sufficient content validity (any level) and at least low quality evidence for sufficient internal consistency. Category C includes PROMs with high quality evidence for an insufficient measurement property. PROMs which cannot be categorized as either A or C are put into category B. The PROMs categorized as “A” are recommended for use while those categorized as “C” are not recommended. PROMs categorized as “B” have potentials to be recommended, but further studies are needed to assess their qualities [16].

Results of the present review show that CFQ (Croatian or Albanian) [43, 44] can meet the inclusion criteria of category A, while Subset of the Oral Health Impact Profile (Subset-OHIP) [40] and QMFQ [42] are in category C. The other PROMs are categorized into B. Thus, only CFQ (Croatian or Albanian) is recommended for use. The PROMs in category B can be further divided into two sub-categories according to the rating of hypothesis testing for construct validity. Hypothesis testing for construct validity refers to the extent of subjective assessment related to other measures that are consistent with theoretical measurement construct [25, 59]. If the hypothesis testing of a PROM is rated as sufficient, it can be re-classified into category B1 and has the potential to be recommended for use. Otherwise, if the hypothesis testing of a PROM is rated as insufficient or indeterminate, it will be categorized into B2 and will need further research to assess its quality. Results of the present review show that FIQ-Japanese [7, 33], new-FIQ-Japanese [8], PDC-Tanzania [34], CFQ-Chinese [30], and SMDOA [41] can be classified into category B1, while ICA-1990 [32], PDC-Sudan [26], Index of Eating Difficulty (IED) [35], CFQ-Japanese [36], Food Intake Ability Questionnaire (FIAQ) [37], Masticatory Ability assessment for the Community-dwelling Elderly (MACE) [27] and Masticatory Problem Index (MPI) [39] are in the category B2. Although, Food Intake Ability Questionnaire-key food version (FIAQ-key food version) [37] and Self-reported Masticatory Function (SMF) [45, 46] may be classified as B1, these two PROMs need more research to fully assess their quality. It is difficult to classify FIQ-Chinese [28, 38] into B1 or B2 because the convergent validity was rated as insufficient though its discriminative validity was rated as sufficient. Further studies on this PROM are needed.

In addition to the above-mentioned measurement properties, feasibility and interpretability of PROMs should also be considered when making recommendations for use [17]. Feasibility refers to the ease of PROM application, such as completion time and cost, while interpretability refers to the relationship between PROM scores and clinical meaning [48]. Considering all the evaluated properties of the PROMs included in the present review, CFQ (Croatian or Albanian) is recommended for use, and FIQ-Japanese, new-FIQ-Japanese, PDC-Tanzania, CFQ-Chinese and SMDOA have the potential to be recommended for use.

Based on the results of this systematic review, none of the included PROMs can be considered as the “gold standard”. Nevertheless, some PROMs have better psychometric properties than others, and may be suitable for certain populations. Specially, CFQ (Croatian or Albanian) is recommended to be used to assess masticatory function of general prosthodontic patients. FIQ-Japanese and New-FIQ-Japanese may be recommended to assess masticatory function of complete denture wearers. SMDOA, CFQ-Chinese and PDC-Tanzania may be recommended to assess masticatory function of community-dwelling adults in epidemiological screening.

To the best of our knowledge, this is the first systematic review on PROMs for masticatory function based on the COSMIN guideline. In addition, this review provides recommendations for the selection of appropriate PROMs for masticatory function. Moreover, this review points out the commonly neglected methodological aspects among the included studies and provides suggestions for future research. Regarding the limitations of the present review, only articles published in English were included and this may result in omission of potentially excellent PROMs reported in articles published in non-English languages. Besides, the PROM development or validation processes may have been rigorously implemented in some studies but were not reported in detail, which may lead to a downgrade of their methodological quality ratings. It is strongly recommended that future studies refer to the COSMIN guideline when developing or validating PROMs.

## Conclusions

Currently, there is no PROM for masticatory function in adults with high-level evidence on all the psychometric properties. There are variations in the psychometric properties among the different reported PROMs. Within the limitations and current evidence of this systematic review, CFQ (Croatian or Albanian), FIQ-Japanese, new-FIQ-Japanese, CFQ-Chinese, SMDOA and PDC-Tanzania outperform other measurement tools. However, well-designed studies on PROMs are needed in the future.

## Supplementary Information

**Additional file 1:** Search strategy, articles from the supplementary search, and details about reliability and validity assessments of the included PROMs.

## Data Availability

All data generated and analyzed in this review are included in the articles.

## Abbreviations

PROMs: Patient-Reported Outcome Measures
COSMIN: COnsensus-based Standards for the selection of health Measurement INstruments
