# Supplementary material for: Patient-reported outcome measures for masticatory function in adults: a systematic review
Source: BMC Oral Health. 2021 Nov 23;21:603. doi: 10.1186/s12903-021-01949-7 (PMC8609720; doi:10.1186/s12903-021-01949-7)
Supplement: Supplementary file 1 — Additional file 1: Search strategy, articles from the supplementary search, and details about reliability and validity assessments of the included PROMs. [file 12903_2021_1949_MOESM1_ESM.docx]

**Additional File**

**Part 1**

**Medline via Pubmed (March 14th, 2021) - 883**

#1 (Mastication[MeSH Terms]) OR Bite force[MeSH Terms]

#2 (((masticat*[Title/Abstract]) OR ("oral function"[Title/Abstract])) OR ("bite force"[Title/Abstract])) OR (chew*[Title/Abstract])

#3. #1 OR #2

#4 (((((Surveys and Questionnaires[MeSH Terms])) OR Patient Health Questionnaire[MeSH Terms]) OR Diagnostic Self Evaluation[MeSH Terms]) OR Self-Assessment[MeSH Terms]) OR Patient Outcome Assessment[MeSH Terms]

#5 (questionnaire* [Title/Abstract]) OR subjective*[Title/Abstract] OR evaluation* [Title/Abstract] OR assessment* [Title/Abstract]

#6. #4 OR #5

#7 ((Reproducibility of Results[MeSH Terms]) OR Validation Studies as Topic[MeSH Terms]) OR Psychometrics[MeSH Terms]

#8 (((((reproducible[Title/Abstract]) OR reproducibility[Title/Abstract]) OR reliable[Title/Abstract]) OR reliability[Title/Abstract]) OR valid*[Title/Abstract]) OR psychometric*[Title/Abstract]

#9. #7 OR #8

#10. #3 AND #6 AND #9

**Embase via Ovid (March 12nd, 2021)-793**

1. exp mastication/

2. (masticat* or oral function or bite force or chew*).ab.

3. 1 or 2

4. *questionnaire/

5. *patient health questionnaire/

6. *self-evaluation/

7. *outcome assessment/

8. (questionnaire* or subjective* or evaluation* or assessment*).ab.

9. 4 or 5 or 6 or 7 or 8

10. exp reliability/

11. exp validity/

12. *psychometry/

13. (reproducible or reproducibility or reliable or reliability or valid* or psychometric*).ab.

14. 10 or 11 or 12

15. 3 and 9 and 14

**CINAHL Plus via EBSCOhost (March 14th, 2021)-264**

S1. MH mastication OR MH Bite force

S2. TX masticat* OR TX chew* OR TX “bite force” OR TX “oral function”

S3. S1 OR S2
S4. MH ( surveys and questionnaire ) OR MH patient health questionnaire OR MH diagnostic self evaluation OR MH patient outcome assessment OR MH self assessment

S5. TX questionnaire* OR TX subjective* OR TX evaluation* OR TX assessment*

S6. S4 OR S5

S7. MH reproducibility of results OR MH validation studies OR MH psychometrics

S8. TX reproducible OR TX reproducibility OR TX valid* OR TX psychometric* OR TX reliable OR TX reliability

S9. S7 OR S8

S10. S3 AND S6 ANS S9

**Web of Science Core Collection (March 14th, 2021)-1050**

TOPIC: (masticat* OR “oral function” OR “bite force” OR chew*)

AND

TOPIC: (questionnaire* OR subjective* OR evaluation* OR assessment*)

AND

TOPIC: (reproducible OR reproducibility OR reliable OR reliability OR valid* OR psychometric*)

**APA PsycINFO (ProQuest) (March 14th, 2021)-67**

1. MAINSUBJECT.EXACT.EXPLODE("Chewing ") OR ab(masticat*) OR ab(“oral function”) OR ab(“bite force”) OR ab(chew*)

2. (MJMAINSUBJECT.EXACT("Questionnaires ") OR MJMAINSUBJECT.EXACT("Self-Evaluation") OR (ab(questionnaire*) OR ab(subjective*) OR ab(evaluation*) OR ab(self-assessment*) OR ab(assessment*))
3. (ab(reproducible) OR ab(reproducibility) OR ab(reliable) OR ab(reliability) OR ab(valid*) OR ab(psychometric*)) OR ((MJMAINSUBJECT.EXACT("Test Validity") OR MJMAINSUBJECT.EXACT("Statistical Validity")) OR (MJMAINSUBJECT.EXACT("Test Reliability") OR MJMAINSUBJECT.EXACT("Statistical Reliability")) OR MJMAINSUBJECT.EXACT("Psychometrics")

4. 1 AND 2 AND 3

**Part 2. Articles from the supplementary search**

Hirai T, Ishijima T, Koshino H, Anazai T. 1994. Age-related change of masticatory function in complete denture wearers: Evaluation by a sieving method with peanuts and a food intake questionnaire method. Int J Prosthodont. 7(5):454–460.

Hsu KJ, Lee HE, Lan SJ, Huang ST, Chen CM, Yen YY. 2012. Evaluation of a self-assessed screening test for masticatory ability of Taiwanese older adults. Gerodontology. 29(2):1–8.

*Khalifa N, Allen PF, Abu-Bakr NH, Abdel-Rahman ME. 2013. Chewing ability and associated factors in a Sudanese population. J Oral Sci. 55(4):349–357.

Koshino H, Hirai T, Toyoshita Y, Yokoyama Y, Tanaka M, Iwasaki K, Hosoi T. 2008. Development of New Food Intake Questionnaire Method for Evaluating the Ability of Mastication in Complete Denture Wearers. Prosthodont Res Pract. 7(1):12–18.

Leake JL. 1990. An Index of Chewing Ability. J Public Health Dent. 50(4):262–267.

*Miura H, Sato K, Hara S, Yamasaki K, Morisaki N. 2013. Development of a Masticatory Indicator Using a Checklist of Chewable Food Items for the Community-Dwelling Elderly. ISRN Geriatr. 2013:1–4.

*Montero J, Dib A, Guadilla Y, Blanco L, Flores J, Gómez-Polo C. 2020. Responsiveness of the different methods for assessing the short-term within-subject change in masticatory function after conventional prosthetic treatments. J Prosthet Dent. 123(4):602–610.

Sarita PTN, Witter DJ, Kreulen CM, Van’t Hof MA, Creugers NHJ. 2003. Chewing ability of subjects with shortened dental arches. Community Dent Oral Epidemiol. 31(5):328–334.

Sato Y, Minagi S, Akagawa Y, Nagasawa T. 1989. An evaluation of chewing function of complete denture wearers. J Prosthet Dent. 62(1):50–53.

Tsuga K, Carlsson GE, Österberg T, Karlsson S. 1998. Self‐assessed masticatory ability in relation to maximal bite force and dental state in 80‐year‐old subjects. J Oral Rehabil. 25(2):110–116.

Yanagisawa T, Ueno M, Shinada K, Ohara S, Kawaguchi Y. 2010. Validity of Self-reported Masticatory Function in a Japanese Population. Japanese Soc Dent Heal. 60:214–223.

Note: Articles with “*” before the article information are included from Google Scholar, and other articles are included through manual search of references of included articles or relevant reviews.

**Part 3. Details about reliability and validity assessments of the included PROMs.**

| **PROMs** | **Internal Consistency** | **Test-retest Reliability** | **Content validity** | **Structural validity** | **Criterion validity** | **Hypothesis testing for construct validity** | **Responsiveness** | **Cross-cultural translation/validity** |
| --- | --- | --- | --- | --- | --- | --- | --- | --- |
| CFS | 0 | 0 | 0 | 0 | 0 | 0 | 0 | 0 |
| ICA-1990 | Reproducibility: 0.98; scalability: 0.68 | 0 |  | 0 | 0 | Convergent validity: compared with two other measures of chewing ability, sensitivity: 0.29-0.66, specificity: 0.80-0.91, positive predictive value: 0.25-0.42, negative predictive value: 0.91-0.92. | 0 | 0 |
| ICA-2020 | 0 | 0 | 0 | 0 | 0 | 0 | Effect size with difficulty masticating 5 different types of food (ES: 0.7 to 1.3). | 0 |
| FIQ-Japanese-1994 | 0 | 0 | 0 | 0 | 0 | Convergent validity: correlation with masticatory performance using sieving method (r = 0.83, *P* < 0.01). | 0 | 0 |
| FIQ-Japanese-1998 | 0 | 0 | 0 | 0 | 0 | Convergent validity: Correlation with maximum bite force (r = 0.532, *P* < 0.01). | 0 | 0 |
| New-FIQ-Japanese | Cronbach’s α: 0.939 | 0 | 0 | Factor analysis, 5 factors explained 66.7% of the variance. | Short version of previous questionnaire, correlation with original mastication score (r = 0.95, *P* < 0.01). | Convergent validity: correlation with masticatory performance using sieving method (r = 0.62, *P* < 0.01). | 0 | 0 |
| FIQ-Chinese-2012 | Cronbach’s α: 0.830 for 23 food groups | 30 patients;  1 weeks; spearman’s rank correlation coefficient (0.801) for 23 food groups | 0 | 0 | 0 | Discriminant validity: AUC value was 0.8294, indicating excellent discriminator. | 0 | 0 |
| FIQ-Chinese-2014 | Cronbach’s α: 0.881, 0.869-0.883. | 30 elders; 1 weeks; spearman’s rank correlation coefficient (0.801) (p<0.001) | 0 | 0 | 0 | Convergent validity: correlation with self-perceived masticatory ability (r = 0.434, P< 0.0001). | 0 | 0 |
| PDC-Tanzania | Cronbach’s α: 0.88 (hard food), 0.93 (soft food) | 0 | 0 | 0 | 0 | Discriminant validity: significant differences about the PDC score between different dental arch categories (*P* < 0.05). | 0 | 0 |
| PDC-Sudan | Cronbach’s α: 0.89 | 20 patients;  2 weeks; ICC: 0.78-0.96 | 0 | 0 | 0 | Convergent validity: correlation with chewing complaints, OHIP-14, FTU anterior and FTU posterior (P < 0.05). | 0 | 0 |
| IED | Reproducibility coefficient: 0.99; scalability coefficient: 0.89 | 106 subjects; 1 week; weighted kappa: 0.89 | Unclear information. | 0 | 0 | Convergent validity: correlation with Chinese versions of General Eating Difficulty (r = 0.59, *P* < 0.001), Dissatisfaction with chewing ability (r = 0.45, *P* < 0.001) and Oral impact on daily performances eating score (r = 0.36, *P* < 0.001). | 0 | 0 |
| CFQ-Japanese | Cronbach’s α: 0.90 | 62 subjects;  2 weeks; ICC: 0.69, 0.56-0.82 | 0 | EFA, 2 factors explained 74% of the variance. | 0 | Convergent validity: correlation with OHIP-14 summary score (r = -0.46, *P* < 0.001) and the number of teeth (r = 0.34). | 0 | 0 |
| CFQ-Chinese | Cronbach’s alpha: 0.912 | Weighted kappa: 0.6-1; agreement of responses: 77.8%-100%. | Comprehensibility was evaluated by participant; relevance and comprehensiveness was evaluated in the professional’s perspectives. | Factor analysis, unidimensional, one factor explained 56.5% of the variance. | 0 | Convergent validity, correlation with two objective assessment methods (rho = 0.515, p < 0.001; rho = 0.663, p < 0.001) and two global self-rated chewing ability questions (rho = 0.617, p < 0.001; rho = 0.562, p < 0.001). Discriminant validity, higher objective result with higher CFQ total scores (p < 0.001) and higher median scores of individual food items (p < 0.005). | 0 | 0 |
| FIAQ | 0 | 0 | 0 | Factor analysis, 4 factors, unclear information. | 0 | 1, Convergent validity： correlation with bite force (r = 0.45, *P* < 0.01).  2, Discriminant validity: the FIA-30 score and FIA-5 score showed a similar discriminating ability. | 0 | 0 |
| FIAQ-key food version | 0 | 0 | 0 | 0 | Cluster analysis: the FIA-30 score  and FIA-5 score showed a similar discriminating ability of three masticatory function groups. | 1, Convergent validity: correlation with bite force (r = 0.51, *P* < 0.01).  2, Discriminant validity: the FIA-30 score and FIA-5 score showed a similar discriminating ability. | 0 | 0 |
| MACE | Cronbach’s α: 0.89 | 0 | 0 | 0 | Correlation with mastication score (r = 0.90, *P* < 0.001). | Convergent validity: correlations with GOHAI (r = 0.48, *P* < 0.001) and the number of teeth (r = 0.40, *P* < 0.001). | 0 | 0 |
| MPI | 0 | 0 | 0 | 0 | 0 | Convergent validity: correlation with Eichner index (r = 0.25, *P* < 0.001), number of teeth (r = -0.27, *P* < 0.001) and maximum bite force (r = -0.21, *P* <0.01). | 0 | 0 |
| Subset-OHIP | Cronbach’s α: 0.803 | 0 | 0 | 0 | 0 | Convergent validity: correlation with measured masticatory efficiency by sieve method (r = -0.14, *P* = 0.22). | 0 | 0 |
| SMDOA | 0 | 0 | Assume relevant, CVI: 0.86. | CFA, unidimensional, 2 factors extracted, correlation between factors f1 and f2 was classified as strong (Φ = 0.83; SE = 0.04). CFI, 0.98; TLI, 0.98; RMSEA, 0.05. | 0 | Discriminant validity: by comparing the CFA of the two-factor model with the same model by a factor. A better fit quality was verified in the distribution of the model in two factors. | 0 | 0 |
| CFQ-Croatian | Cronbach’s α: 0.916 | 60 participants;  2 weeks; ICC: 0.991, 0.945-0.985 | Unclear information | Factor analysis, unidimensional, one factor explained 63.67% of the variance. | 0 | 1, Convergent validity: significant positive association with the general single question (r = 0.628, *P* < 0.001) and the OHIP summary score questions related to chewing (r = 0.721, *P* < 0.001).  2, Discriminant validity: significant difference between the NT group and the RDWs group (*P* < 0.001). | 24 participants; paired t-test and standardized effect size.  Mean change score was 10.67 (SD = 8.47) (*P* < 0.001) and standardized effect size was 0.95. | 0 |
| CFQ-Albanian | Cronbach’s α: 0.974 | 61 subjects;  2 weeks; ICC: 0.90, 0.43-0.81 | Unclear information | EFA, one-dimensional model, one-factor explained 81.711 % of the variance. | 0 | 1, Convergent validity: correlation with self-reported general satisfaction with chewing ability (r = 0.884, *P* < 0.01).  2, Discriminant validity: scores differentiated between all sample groups: with natural teeth, fixed partial dentures and removable denture wearers (*P* < 0.001). | 51 prosthodontic patients; paired t-test and standardized effect size.  Mean change score was 15.57 (SD = 2.49) (*P* < 0.001) and effect size was 2.03. | For cross-cultural translation:  1, Forward-backward translation by independent translators.  2, Pilot study. |
| QMFQ-Persian | Cronbach’s α: 0.914 | 0 | Translation of original scale, assume relevant, evaluated by four experienced prosthodontists. | EFA, 5 domains explained 77.6% of the variance. | 0 | Convergent validity: correlation with the years of edentulism (r = -0.417, *P* < 0.001). | 0 | For cross-cultural translation: Forward-backward translation. |
| SMF-Yanagisawa | 0 | 0 | 0 | 0 | 0 | Convergent validity: correlation with mean number of total and posterior teeth (r = 0.797, *P* < 0.001; r = 0.754, *P* < 0.001); correlation with n-FTUs, nif-FTUs and total-FTUs (r = 0.701, *P* < 0.001; r = 0.689, *P* < 0.001; r = 0.651, *P* < 0.001). | 0 | 0 |
| SMF-Ueno | 0 | 0 | 0 | 0 | 0 | Convergent validity: >75% of the results in accordance with hypothesis. Relationship with n-FTUs, nif-FTUs and total-FTUs (*P* < 0.001). | 0 | 0 |

Note: 0 = no data available.
